# Low-Velocity Impact Behavior of PLA BCC Lattice Structures: Experimental and Numerical Investigation with a Novel Dimensionless Index †

**DOI:** 10.3390/ma18194574

**Published:** 2025-10-01

**Authors:** Giuseppe Iacolino, Giuseppe Mantegna, Emilio V. González, Giuseppe Catalanotti, Calogero Orlando, Davide Tumino, Andrea Alaimo

**Affiliations:** 1 Department of Engineering and Architecture, Kore University of Enna, Cittadella Universitaria, 94100 Enna, EN, Italy; giuseppe.mantegna@unikore.it (G.M.); giuseppe.catalanotti@unikore.it (G.C.); calogero.orlando@unikore.it (C.O.); davide.tumino@unikore.it (D.T.); andrea.alaimo@unikore.it (A.A.); 2 AMADE, Polytechnic School, University of Girona, Campus Montilivi s/n, 17071 Girona, Spain; emilio.gonzalez@udg.edu

**Keywords:** lattice structures, numerical and experimental comparison, drop-weight impact test, PLA, dimensionless index, additive manufacturing

## Abstract

Lattice structures are lightweight architected materials particularly suitable for aerospace and automotive applications due to their ability to combine mechanical strength with reduced mass. Among various topologies, Body-Centered Cubic (BCC) lattices are widely employed for their geometric regularity and favorable strength-to-weight ratio. Advances in Additive Manufacturing (AM) have enabled the precise and customizable fabrication of such complex architectures, reducing material waste and increasing design flexibility. This study investigates the low-velocity impact behavior of two polylactic acid (PLA)-based BCC lattice panels differing in strut diameter: BCC1.5 (1.5 mm) and BCC2 (2 mm). Experimental impact tests and finite element simulations were performed to evaluate their energy absorption (EA) capabilities. In addition to conventional global performance indices, a dimensionless parameter, $D^{*},$ is introduced to quantify the ratio between local plastic indentation and global displacement, allowing for a refined characterization of deformation modes and structural efficiency. Results show that BCC1.5 absorbs more energy than BCC2, despite the latter’s higher stiffness. This suggests that thinner struts enhance energy dissipation under dynamic loading. Despite minor discrepancies, numerical simulations provide accurate estimations of EA and support the robustness of the $D^{*}$ index within the examined configuration, highlighting its potential to deformation heterogeneity.

## 1. Introduction

Cellular solids represent a significant category of engineering materials. Due to their superior properties, these structures are a promising solution for various industrial applications [1]. They offer a unique combination of lightweight design and mechanical strength, making them particularly beneficial in fields that demand high performance with reduced weight, such as the aerospace and automotive industries [2]. As reported in [3], cellular solids can be divided into two categories: foams and lattice structures, both characterized by their lightweight properties. Foams, which can also be metallic, possess excellent heat dissipation, thermal and electrical conductivity [4]. On the other hand, lattice structures possess high specific strength and stiffness, and optimal structural properties [2,4]. As reported in [5], within the macro area of lattice structures, three additional categories can be identified: disordered, periodic, and pseudo-periodic lattice structures. Specifically, the first category is characterized by unit cells that are arranged randomly with varying cell sizes. The second category, periodic lattice structures, refers to the periodic repetition of lattice cells that have specific shapes and topology within three-dimensional Euclidean space. Conversely, the third category, pseudo-periodic or conformal lattice structures [2], is characterized by each unit cell maintaining a consistent topology while varying in size. Furthermore, among periodic lattice structures, there are various types, such as Diamond [6], Face-Centered Cubic and Body-Centered Cubic lattices (BCC) [7]. However, BCC ones represent one of the most common configurations and are the focus of the present study, not only for their well-defined geometry but also for their extensively studied mechanical behavior. In detail, BCC lattices are typically bending-dominated [8], a behavior resulting from the limited number of struts per unit cell, which leads to load transfer primarily through bending rather than axial forces. This results in a relatively higher specific energy absorption (SEA) [9] under deformation. Furthermore, to estimate their effective mechanical properties based on the bulk material and relative density, the Gibson–Ashby model [3] is commonly adopted, providing a theoretical framework widely used in the analysis of lattice materials.

From a manufacturing standpoint, various techniques can be employed. Noteworthy methods include the creation of perforated sheets [10], and selective laser melting [11], with comprehensive reviews available in [12,13].

The industrial application of lattice structures is especially esteemed due to the capability to incorporate advanced production technologies such as additive manufacturing (AM). According to [14,15], AM technologies provide the capability to create custom-designed components with complex geometries while minimizing material waste. It allows the use of a wide range of materials, including metals [16,17], and composites [18,19]. Furthermore, numerous studies in the literature have explored the application of polymeric materials. Among these, particular attention is given to those in which polylactic acid (PLA) is used, as in the present study. In particular, BCC lattices manufactured with PLA have been widely adopted in energy absorption (EA) applications, due to their ability to sustain large deformations while maintaining general structural integrity. For instance, in [20] the authors investigated the low-velocity impact behavior of bi-material structures composed of 3D-printed PLA lattice frames impregnated with polyurethane foams. The use of PLA provided a lightweight yet sufficiently strong structural framework for evaluating the effect of different foam reinforcements. Similarly, [21] employs PLA as the base material for additively manufactured lattice structures tested under low-velocity impact. Three different core configurations were investigated—honeycomb, tubular, and truss—with the truss design exhibiting the highest specific EA under identical impact conditions. Furthermore, Hedayati et al. [22] employed PLA as the base material for AM-fabricated lattice sandwich panels subjected to low-velocity impact. The study highlights PLA’s suitability for lightweight energy-absorbing structures, while also addressing its sensitivity to environmental degradation affecting impact performance.

Based on the above, the use of PLA in this study represents a consistent choice with the current state of the art, which highlights this material for its lightweight nature and good printability. Moreover, PLA was selected due to its wide commercial availability, low cost, ease of processing, and mechanical properties suitable for the impact conditions investigated. Thus, AM represents a significant advancement in the field of production and, as already mentioned, offers numerous advantages. To this end, the present study investigates two additively-manufactured PLA-BCC lattice configurations, differing only in strut diameter (1.5 mm and 2 mm). Since the local impact response of lattice structures depends on the specific point of impact, this research addresses a gap in the literature by proposing a method to quantify localized deformation under low-velocity impacts—an aspect that conventional global metrics such as EA, SEA, or mean crushing force (MCF) fail to capture. In this regard, a novel dimensionless index, $D^{*}$, capable of capturing the interplay between local plastic indentation and global displacement, is introduced. In each configuration, the independent variable is the strut diameter, while the dependent properties include EA, MCF, SEA, and the newly proposed $D^{*}$ index. Both experimental drop-weight impact tests and finite element simulations were conducted, allowing for a comprehensive comparison and validation of the proposed metric across physical and numerical domains. This dual approach provides a robust framework to evaluate how geometry-driven local effects influence global EA capacity in lattice structures.

This work is organized into the following sections: Section 2 introduces the theoretical background and defines the aforementioned index for both experimental and numerical cases. Section 3 describes the experimental and numerical setup. Section 4 presents the results. Section 5 presents a comparison between numerical and experimental results and discusses the effectiveness of the proposed index, while the conclusions are reported in Section 6.

## 2. Theoretical Background

### 2.1. Specific Energy Absorption and Mean Crushing Force

A key parameter in the analysis of impact behavior is the EA [23], which is defined by the following expression:

(1) $$EA\left[J\right]=\int_{0}^{\delta_{max}}F\left(\delta \right)d\delta$$

In Equation (1), $\delta_{max}$ stands for the total global displacement, and F(δ) represents the impact force during crushing. Furthermore, the mechanical performance and EA capacity can be assessed using the SEA, the MCF, and the Peak Crushing Force ($F_{max}$) [24].

The SEA is defined in Equation (2):

(2) $$SEA\left[\frac{J}{g}\right]=\frac{EA}{m}$$

where *m* is the mass of the structure before the impact. Furthermore, the MCF can be defined as:

(3) $$MCF\left[\frac{J}{mm}\right]=\frac{EA}{\delta_{max}}$$

Finally, the $F_{max}$ is the maximum force recorded on the displacement–force curve. To ensure occupant safety during impact, the $F_{max}$ value must remain below a specified threshold.

While the aforementioned indices quantify the global crash response, they do not encode the spatial distribution of plastic strain. EA, MCF, and $F_{max}$ depend on material, geometry, boundary conditions, and impact velocity. SEA is still insensitive to how deformation is distributed and thus cannot distinguish localized from diffuse damage. This limitation has motivated some studies to propose impact-specific indices, each focusing on different aspects of the structural response. For instance, [25] defines a dimensionless scalar that captures the lateral asymmetry of localized damage caused by oblique impacts, normalized by the global indentation depth. Similarly, [26] introduces a set of dimensionless numbers, known as the DLV (Density–Length–Velocity) system, designed to represent the scaled behavior of structures under impact loading. Despite the valuable contributions and to the best of the authors’ knowledge, a gap persists in the development of indices specifically tailored to quantify localized deformation with respect to the overall structural response. This need is relevant in the context of AM, where additively-manufactured components are increasingly employed in applications involving impact loading conditions.

This consideration motivated the development of a new dimensionless local deformation index, $D^{*}$, specifically designed to aggregate the effects of localized indentations into a single scalar value, normalized by the observed overall structural response. The following subsection details the procedure used to compute this index for both experimental tests and numerical simulations of low-velocity drop-weight impacts.

### 2.2. D^*^ Index for Experimental and Numerical Tests

In its general form, the index is defined as the mean of the two dent depths $d_{1}$ and $d_{2}$ which represent a quantitative experimental measure of localized damage, as suggested in [27]. The mean is denoted with $\bar{d}$. The resulting value is then normalized by the experimental maximum global displacement $\delta_{max}$ as shown in Equation (4).

(4) $$D^{*}=\frac{\bar{d}}{\delta_{max}},\delta_{max}>0$$

**Figure 1:**
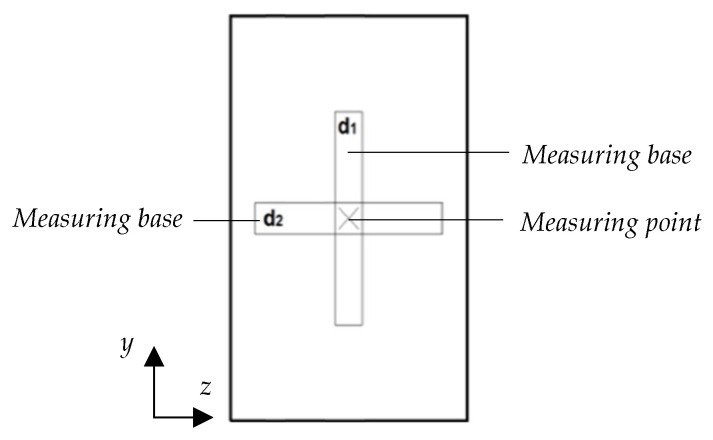
Dent depth $d_{1}$ and $d_{2}$.

This provides a compact, dimensionless measure of the intensity of local plastic deformation relative to the global maximum displacement. The values of $d_{1}$ and $d_{2}$ are obtained after each experimental impact test using a depth gauge applied to the nominally flat impacted point, ensuring proper seating of the measuring device. To perform the measurements, the measuring base is repositioned twice on the impacted area, as illustrated in Figure 1. Specifically, $d_{1}$ and $d_{2}$ are evaluated because the surface may exhibit local irregularities and roughness arising from the AM process. Since the probe tip remains fixed while only the measuring base is repositioned, both measurements correspond to the same physical point. To acquire the $d_{2}$, the measuring base is positioned along the *z*-axis. Subsequently, to obtain the $d_{1}$ value, the measuring base is rotated and aligned along the *y*-axis. These values, despite being negative, are reported as positive as the sign is determined solely by the chosen reference system. This setup allows the probe tip to reach and contact the deepest point formed by the impact.

In the case of numerical simulations, only a single value of dent depth $d\;=\bar{d}$ is available since the impacted surface is geometrically regular and flat ($d_{1}=d_{2}$). Thus, no thickness variations or surface irregularities could affect the indentation depth along different directions. The displacement is extracted from the central node on the top surface of the specimen, corresponding to the same physical location probed during the experimental measurements. In the numerical context, the global displacement $\delta_{max}$ is recorded at the time instant corresponding to the peak impact force, while the local indentation depth d is evaluated at the end of the simulation, when the impactor has rebounded or come to rest. Furthermore, according to Equations (2) and (3), Equation (4) can be reformulated in:

(5) $$D^{*}=\frac{MCF*\bar{d}}{SEA*m}$$

This formulation, valid for both experimental and numerical results, clarifies the relationship between $D^{*}$ and the physical quantities involved in its definition. Specifically, Equation (5) shows that $D^{*}$ increases when $MCF*\bar{d}$ grows faster than SEA∗m. Conversely, $D^{*}$ decreases when SEA∗m dominates the contribution from local deformation and average force.

## 3. Materials and Methods

### 3.1. Experimental Setup and Statistical Analysis of the Samples

In this study, two configurations were investigated, BCC1.5 and BCC2, with six specimens tested for each category. The key difference between the two configurations lies in the strut diameter Φ: BCC1.5 features Φ = 1.5 mm, whereas Φ = 2 mm for BCC2.

All specimens, fabricated from PLA, exhibit the mechanical properties detailed in Table 1. These include the elastic modulus (*E*), which describes the stiffness of the material in its elastic regime, and the yield strength ($\sigma_{yield}$), marking the onset of plastic deformation. Alongside these, the table reports the strain at break ($\varepsilon_{break}$), which quantifies the material’s ability to deform before failure, and the ultimate strength ($\sigma_{max}$), indicating the maximum stress sustained before rupture. To obtain these properties, three dog bone specimens were tested under tensile test conditions in accordance with ASTM D638 standards [28], with an average weight of 10.26 g and a standard deviation of 0.5 g.

**Figure 2:**
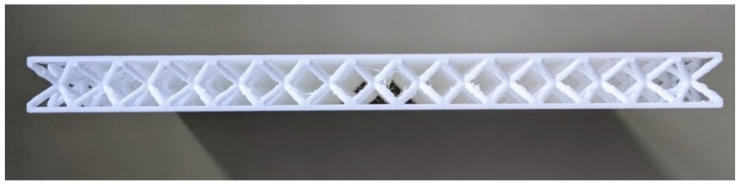
Side view of the BCC1.5 sample exhibiting the lowest quality among the tested specimens.

**Figure 3:**
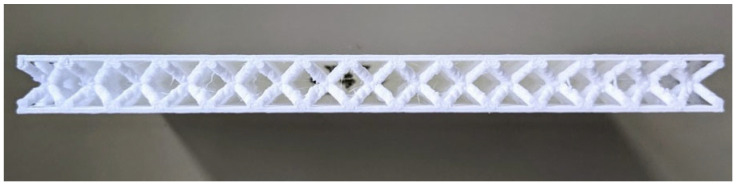
Side view of the BCC2 sample exhibiting the lowest quality among the tested specimens.

To manufacture the samples, the Flashforge (Hangzhou, China) Dreamer 3D printer was employed at the Laboratory of Mechanical and Aerospace Engineering (LIMA) of the Kore University of Enna, Italy. In accordance with ASTM D7136 standards [29], each specimen should ideally have nominal dimensions of 150 mm in length ($L_{nom}$), 100 mm in width ($W_{nom}$), and 12 mm in thickness ($t_{nom}$). This thickness consists of 10 mm for the BCC cell and 1 mm each for the top and bottom skins. The side length *L* of the individual cell measures 10 mm. Additionally, the specimens were tested at the Analysis and Advanced Materials for Structural Design (AMADE) research group at the University of Girona, Spain. A comparison of the side views of two representative BCC1.5 and BCC2 samples is presented in the following Figure 2 and Figure 3, respectively. The samples shown correspond to those with the lowest quality within each category, selected to highlight the most significant manufacturing defects associated with the AM process.

**Figure 4:**
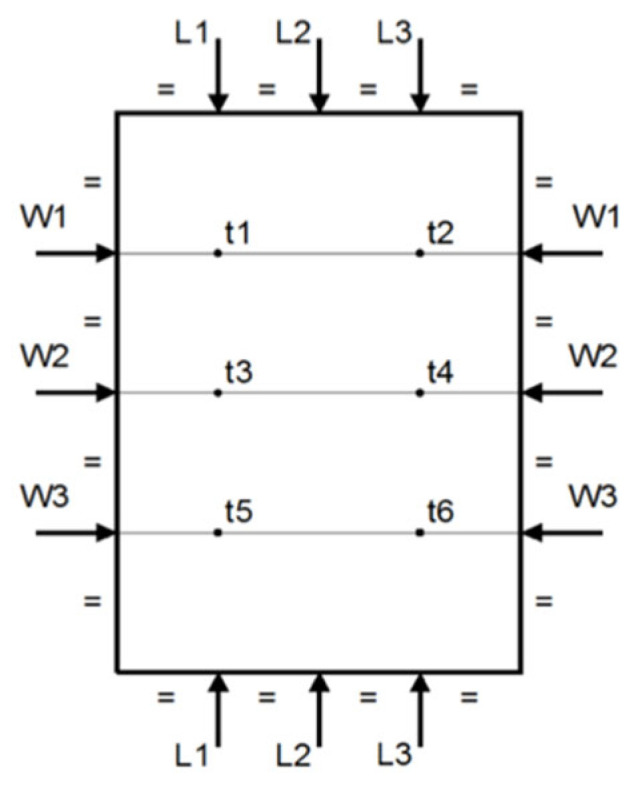
Representation of the points where measurements were taken.

The specimens were tested using the CEAST Fractovis Plus machine (Instron, Norwood, MA, USA), as in the works of Gonzalez et al. [30] and Al-Shamary et al. [31]. In the present experimental tests, a hemispherical impactor with a diameter of 16 mm and a mass of 2 kg was released from a height of 150 mm, resulting in an impact energy of approximately 2.94 J and an impact velocity of about 1.72 m/s. Before each test, appropriate measurements were taken using a digital caliper. Specifically, the widths ($W_{i}$) at various points *i* were measured, along with the lengths ($L_{i}$) and the thicknesses ($t_{i}$). Moreover, an electronic balance was used to measure the mass values. In Appendix A, Table A1, Table A2 and Table A3 provide the width, length, and thickness measurements, while Table A4 shows the mass values. In addition, Figure 4 provides a representation of the points where the measurements were taken.

For each specimen *j*, the mean width $W_{mean}$, length $L_{mean}$, and thickness $t_{mean}$ were computed as shown in Equation (6),

(6) $$X_{mean,j}=\frac{1}{n}\sum_{i=1}^{n}X_{j,i}$$

where X∈{W,L,t}, with n =3 for width and length, and n =6 for thickness. Furthermore, the overall mean values $X_{M}$ for each geometric parameter across all n =6 specimens in each category were calculated as:

(7) $$X_{M}=\frac{1}{N*n}\sum_{j=1}^{N}\sum_{i=1}^{n}X_{j,i}$$

The standard deviation (*SD*) was computed across the six specimen means for each geometrical feature, thereby capturing the variability between specimens. In Equation (8) the coefficients of variation (CV) are estimated as follows:

(8) $$CV_{X}=100*\frac{SD(X_{mean,j})}{X_{M}}$$

Additionally, the relative deviations from nominal values $\Delta X_{nom}$ were calculated for each parameter, according to Equation (9), where all components refer to the experimental measurement procedure.

(9) $$\Delta X_{nom}\left[\%\right]=100*\frac{X_{M}-X_{nom}}{X_{nom}}$$

### 3.2. Numerical Setup

**Figure 5:**
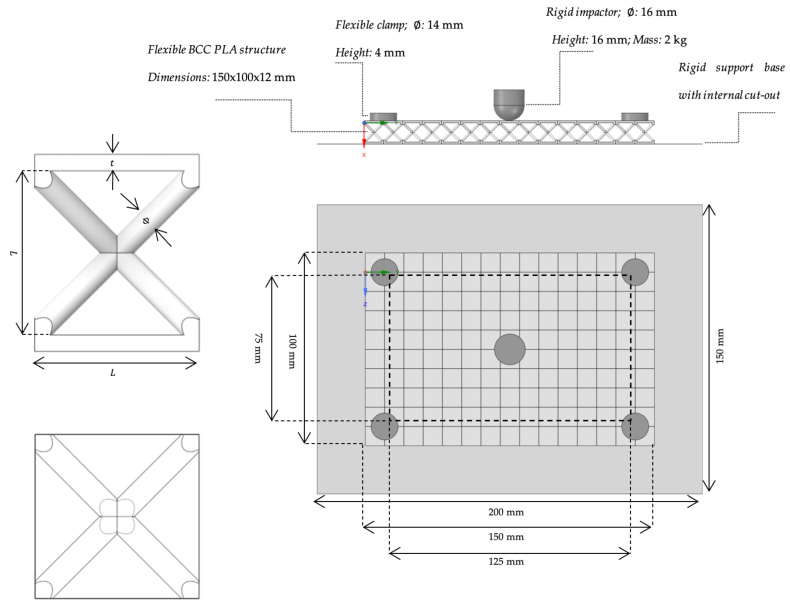
Numerical setup: side view of the single unit (**top left**), translucent top view of the single unit (**bottom left**), side view of the complete setup (**top right**), top view of the complete setup (**bottom right**).

Two numerical simulations were conducted using Ansys LS-DYNA (version 2023 R1) [32]. Figure 5 illustrates the numerical setup, which is identical for both BCC1.5 and BCC2 structures and reproduces the experimental low-velocity impact system described in Section 3.1. The Figure shows the side view of the single unit (top left), the translucent top view of the single unit (bottom left), the side view of the complete setup (top right), and the top view of the complete setup (bottom right). Specifically, the Figure highlights the following components: clamps, impactor, supporting base, and the lattice structure, whether BCC1.5 or BCC2. Both structures are made of PLA, with their mechanical properties provided in Table 1. The BCC1.5 and BCC2 samples have a mass of 56.90 g and 70.39 g, respectively. Additionally, a bilinear material model with a zero tangent modulus was implemented, corresponding to an elastic–perfectly plastic behavior. In this analysis, no material damage model was considered; therefore, cracking or strength degradation are not expected. The material for the impactor was selected to ensure that its geometric characteristics would yield a mass compatible with the experimentally chosen value of 2 kg. As in previous studies such as [33], an artificial density ρ was assigned to the impactor to achieve the desired mass. In this work, a ρ equal to 747 g/$cm^{3}$ was defined.

Furthermore, as in the experimental tests, the impactor was dropped from a height of 150 mm. The support base and the clamps were modeled as rigid and flexible, respectively. All contacts were defined as frictional with a coefficient of friction set to 0.2, which was chosen based on numerical fitting considerations. Moreover, all parts of the model were discretized using four-node linear tetrahedral elements. Given the role of each component in the simulation, the mesh was designed with a variable element size depending on the object. In both simulations, the impactor was meshed with an element size of 2.5 mm, the base with 8 mm, and the clamps with 4 mm. The lattice structure was meshed more finely, with an element size of 0.8 mm for the BCC1.5 and 0.9 mm for the BCC2. The total number of mesh elements was 1,094,012 for BCC1.5 and 836,925 for BCC2. Also, before conducting the analysis, appropriate boundary conditions were defined as follows: the base was fully constrained, a displacement perpendicular to the structure’s upper skin was applied to the impactor, and a force of 50 N was applied to each clamp to prevent any movement of the structure during impact. This clamping force was chosen to avoid bending phenomena on the top skin, which could occur with higher values.

## 4. Results

### 4.1. Dimensional Results

The dimensional analysis reported in Table 2 and Table 3 confirms the good accuracy and repeatability of the AM process. Both BCC1.5 and BCC2 samples exhibit minimal deviations from nominal values in width ($\Delta W_{nom}\leq$ +0.13%) and length (−0.12% $<\Delta L_{nom}<$ +0.20%), while the thickness dimension shows a slightly higher systematic overestimation, particularly in BCC2 (+1.92%). The CV remains consistently low across all dimensions, with values not exceeding 0.31%, highlighting excellent process stability. Notably, BCC1.5 samples show slightly better consistency in width and thickness, whereas BCC2 demonstrates improved repeatability in length. These results indicate that both sample categories are dimensionally reliable and statistically comparable.

Overall, this analysis confirms the effectiveness of 3D printing in producing geometrically precise and consistent lattice structures, with only minor calibration needed for the thickness dimension.

### 4.2. Experimental Drop-Weight Impact Test Results

**Figure 6:**
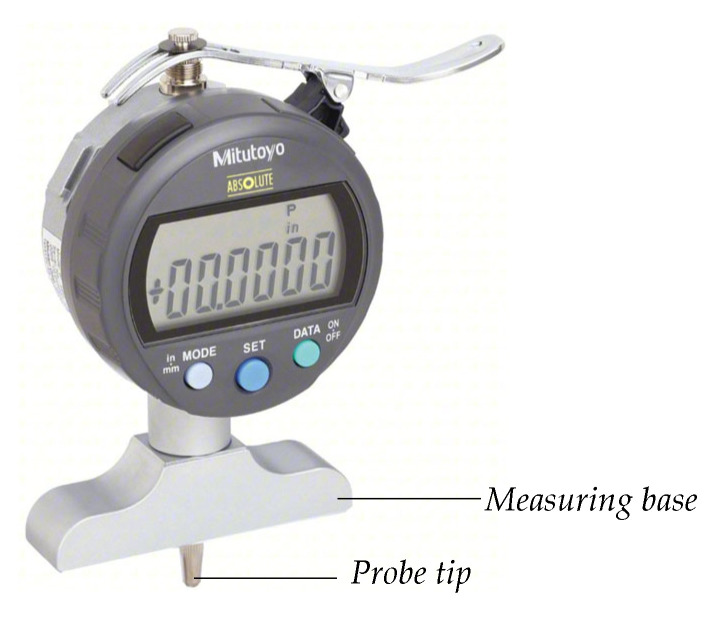
Mitutoyo 547-217S ABS Digital.

In order to acquire the $d_{1}$ and $d_{2}$ measurements, the Mitutoyo series 547-217S ABS Digital, which has an accuracy of ±0.0254 mm [34], depicted in Figure 6, was employed.

**Figure 7:**
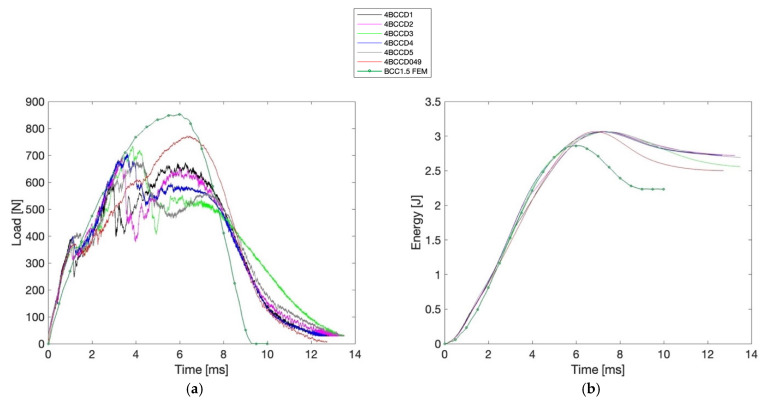

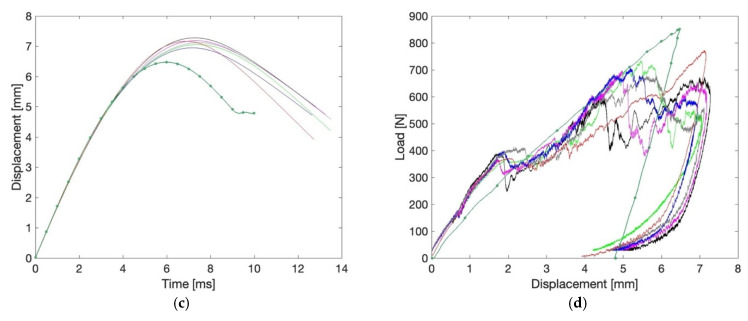
(**a**) Time–Load, (**b**) Time–Energy, (**c**) Time–Displacement, and (**d**) Displacement–Load diagrams for BCC1.5 samples.

**Figure 8:**
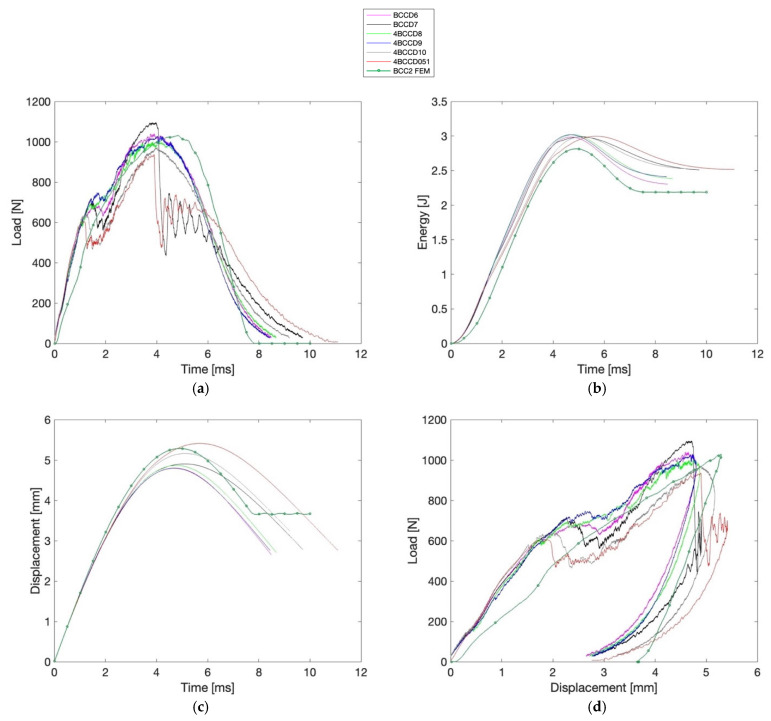
(**a**) Time–Load, (**b**) Time–Energy, (**c**) Time–Displacement, and (**d**) Displacement–Load diagrams for BCC2 samples.

Figure 7 and Figure 8 display the time–load, time–energy, time–displacement, and displacement–load diagrams for the BCC1.5 and BCC2 specimens, respectively, in which the numerical (FEM) curves are also reported.

Additionally, Table 4 and Table 5 report the corresponding values of $F_{max}$, maximum energy ($E_{max}$), $\delta_{max}$, along with $d_{1}$ and $d_{2}$, EA, SEA, MCF and $D^{*}$ values.

As can be observed, the BCC2 configuration shows a $F_{max}$ reaching 1096.00 N, whereas the highest value recorded for BCC1.5 is 772.80 N. Despite the lower force values, BCC1.5 specimens consistently show higher displacement, with a peak of 7.27 mm compared to 5.41 mm in BCC2. The total input energy remains nearly constant across all BCC1.5 samples (3.06 J), while in BCC2 it ranges slightly between 2.98 and 3.01 J. The EA reaches a maximum of 2.72 J in BCC1.5 and 2.53 J in BCC2. The SEA reaches 0.05 J/g in BCC1.5, while BCC2 samples exhibit slightly lower values, with a maximum of 0.03 J/g. Conversely, the MCF attains its highest value in BCC2 (0.51 J/mm), exceeding that of BCC1.5 (0.40 J/mm). Finally, the $D^{*}$ displays a broader range in BCC1.5, with a maximum of 0.84, while in BCC2 the highest value is 0.53. To support these observations, a two-tailed *t*-test was carried out to statistically compare the two groups across all evaluated parameters, with a significance threshold set at p < 0.005. The analysis confirmed statistically significant differences for all indices, except for $d_{1}$ and, consequently, $D^{*}$, where no significant variation was found between the two configurations. In particular, a *p*-value of 0.08 was obtained for $d_{1}$, while $D^{*}$ yielded a *p*-value of 0.36. In Table 6, the experimental values of mean EA, SD EA, $mean\;D^{*}$, and $SD\;D^{*}$ are reported for both BCC1.5 and BCC2 configurations.

At this stage, by correlating $D^{*}$ with the absorbed energy EA, it is possible to assess how much localized indentation mechanisms contribute to the overall EA, independently of the global displacement level. This aspect will be further explored in the following section.

### 4.3. Numerical Drop-Weight Impact Test Results

**Figure 9:**
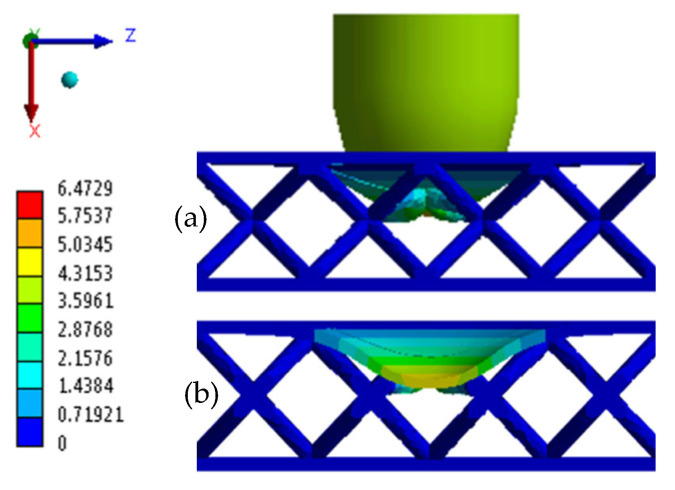
Total Displacement BCC1.5. (**a**) Lateral view; (**b**) section view.

**Figure 10:**
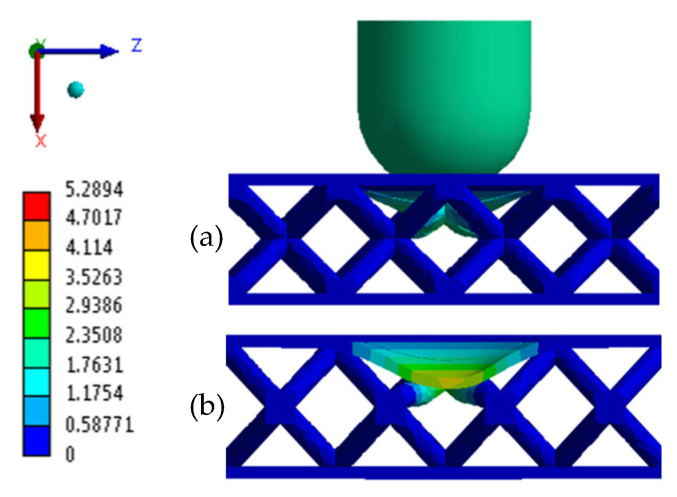
Total Displacement BCC2: (**a**) Lateral view; (**b**) section view.

Following the two numerical simulations, the corresponding results were obtained and are reported in Table 7. For comparative purposes, the table also includes the average experimental values for each category. Furthermore, Figure 9 and Figure 10 report the total displacement for both BCC1.5 and BCC2 structures, respectively.

As observed in Figure 7b and Figure 8b, which overlay the experimental time–energy curves with the corresponding numerical prediction, BCC1.5 exhibits a slightly higher plateau energy (≈2.20 J) than BCC2 (≈2.19 J). In addition, BCC2 demonstrates a greater maximum load of 1030.90 N, exceeding the 852.18 N recorded for BCC1.5. In terms of displacement, BCC1.5 experiences a higher value of 6.47 mm, whereas BCC2 exhibits a lower displacement of 5.29 mm. With regard to the MCF, BCC2 reaches a value of 0.42 J/mm, slightly higher than the 0.35 J/mm observed for BCC1.5. In contrast, the SEA is marginally greater in BCC1.5, with a value of 0.04 J/g compared to 0.03 J/g in BCC2. As for the $D^{*}$ index, BCC1.5 presents a slightly higher value of 0.74, while BCC2 reaches 0.70.

## 5. Discussion

In this work, the dimensional characterization of the PLA-based BCC lattice specimens, manufactured via 3D printing, confirmed high fidelity to the nominal design specifications. Both BCC1.5 and BCC2 samples exhibited deviations below 0.2% for width and length, and thickness increases within acceptable tolerances (+1.2% and +1.9%, respectively). Furthermore, the low coefficient of variation (below 1%) across all measured dimensions highlights the good overall repeatability of the manufacturing process. From a data analysis perspective, Table 4 and Table 5, together with the time–load, time–energy, time–displacement, and displacement–load diagrams presented in Figure 7 and Figure 8, highlight the fundamental influence of strut diameter on the mechanical response of BCC lattice structures under low-velocity impact. The BCC2 configuration, characterized by thicker struts, exhibits significantly higher $F_{max}$—ranging from 937.20 N to 1096.00 N—reflecting an overall stiffer and more rigid structural behavior. However, this increased stiffness limits the structure’s ability to deform, as shown by the lower $\delta_{max}$ observed in BCC2 samples, all below 5.41 mm. In contrast, the BCC1.5 specimens, with thinner struts, reach $\delta_{max}$ values consistently above 7 mm on average, and display a more extended displacement–load curve. These longer deformation paths correspond to higher EA values, calculated according to Equation (1), with BCC1.5 samples averaging 2.65 J compared to 2.44 J for BCC2. This allows the BCC1.5 structures to absorb energy gradually across a larger displacement range according to Table 6. As shown, *SD* EA is identical for both BCC1.5 and BCC2 (0.09 J), indicating a comparable repeatability of the global structural response. The $d_{1}$ and $d_{2}$ dent depth measures further support this interpretation. BCC1.5 specimens exhibit deeper indentations, indicating more significant local plastic deformation. In contrast, BCC2 samples show shallower dents, generally below 3 mm, confirming a more localized and limited response to impact.

Furthermore, a dimensionless index $D^{*}$, defined in Equations (4) and (5), was introduced by providing a simple and physically interpretable metric that quantifies the ratio between local plastic indentation and global displacement. The correlation between $mean\;D^{*}$ and meanEA, also reported in Table 6, suggests that the EA mechanism is not solely governed by $\delta_{max}$ but is also influenced by localized indentation effects. Specifically, this trend can be observed when comparing samples such as 4BCCD2 and 4BCCD5, both belonging to the BCC1.5 category. Despite exhibiting comparable maximum global displacements ($\delta_{max}$ ≈ 7.18 mm and 7.11 mm, respectively), they present different values of $D^{*}$ due to the significantly deeper dent, indicating that localized deformation contributes differently to the overall absorption process. Furthermore, it can be observed that 0.31≲ $D^{*}≲0.84$ for the BCC1.5 specimens, whereas BCC2 sample cluster 0.33 ≲ $D^{*}≲0.53$. SD $D^{*}$ is markedly higher for BCC1.5 (0.19 vs. 0.07), suggesting a greater variability in the localization of plastic deformation, likely due to local instabilities. Moreover, as shown in Table 4, SEA, calculated according to Equation (2), ranges narrowly between 0.04 and 0.05 J/g, and MCF, determined based on Equation (3), between 0.34 and 0.40 J/mm indicating similar EA and average resistance across samples. However, $D^{*}$ spans a wider interval, effectively differentiating between specimens exhibiting distributed versus localized deformation. In our experimental data, values approaching $D^{*}=1$ are not generally associated with a reduction in EA; nonetheless, according to Equation (4), $D^{*}$ flags pronounced perforation for sample 4BCCD5, despite its SEA and MCF values falling within the expected range. This behavior is also evident in the time–load diagrams in Figure 7. Specifically, specimen 4BCCD5 (gray line) clearly stands apart from the others: while all samples display a marked peak between 4 and 8 ms, the response of 4BCCD5 exhibits a pronounced dip in the same time range. A similar pattern holds for BCC2 specimens. Here too, SEA and MCF show little variation (SEA = 0.03 J/g; MCF ≈ 0.48–0.51 J/mm). Yet, specimen 4BCCD051 stands out with a $D^{*}$ of 0.53, indicating a potential early shift toward localization that is not clearly reflected in the global indices. In the time–load diagram, 4BCCD051 corresponds to the red line and displays a curve like that of 4BCCD7 (black line), although it exhibits the lowest peak load among all tested samples.

The numerical data presented in this study show a reasonable agreement with the experimental findings. As reported in Table 7, for the BCC1.5 specimens, the numerical simulation yielded an EA of 2.30 J, an MCF of 0.35 J/mm, and a SEA of 0.04 J/g. These values can be compared to the Mean EXP EA of 2.65 J, Mean EXP MCF of 0.37 J/mm, and Mean EXP SEA of 0.04 J/g, with a percentage error of 13.21% for the EA. Similarly, the BCC2 model returned an EA of 2.25 J, MCF of 0.42 J/mm, and SEA of 0.03 J/g, compared to the Mean EXP values of 2.44 J, 0.49 J/mm, and 0.03 J/g, respectively, corresponding to a percentage error of 7.79% in EA. Nonetheless, the simulations resulted in a $D^{*}$ value of 0.74 for BCC1.5 and 0.70 for BCC2; these can be compared to the ${Mean\;EXP\;D}^{*}$ of 0.46 for BCC1.5, and 0.38 for BCC2.

Because fracture/damage is not explicitly modeled, $D^{*}$ also serves as a potential diagnostic of the deformation pathway—indicating how the computation progressed and the extent to which the model captures expected localization mechanisms. The observed behavior of $D^{*}$ in both experimental and numerical results suggests its relevance not only for interpreting impact responses but also for identifying samples with deformation modes that may compromise structural integrity despite acceptable global metrics.

Undoubtedly, the results obtained depend exclusively on the impact point. This reveals a strong sensitivity of the outcomes to the location of the impact. Therefore, the collected data are valid only when the impactor strikes the standard reference point. In general, for a BCC-type lattice structure, the possible impact scenarios include the impactor striking (i) a node, (ii) between two nodes, or (iii) barycentric among four nodes.

When the impact occurs at one location or another, the global response may remain mostly unaffected, but local variations arise. In particular, the scenario (iii), which corresponds to the configuration adopted in this study, tends to exhibit greater compliance. Impacts in scenario (iii) produce deeper indentations, and thus higher $D^{*}$ values. This is due to the absence of direct energy transfer to the nodes, as most of the impact energy is absorbed by the upper skin. Hence, the findings are not generalizable but rather specific to the analyzed configuration. Consistently with this observation, statistical analysis revealed that $D^{*}$ is not significantly different between the two configurations (p < 0.005). The lack of significance for $D^{*}$ can be attributed to scenario (iii). Under this configuration, $D^{*}$ does not effectively capture the influence of strut diameter differences between BCC1.5 and BCC2. However, $D^{*}$ could become statistically significant when the impact occurs directly on a node, where local deformation is more pronounced. In such cases, the parameter should indeed be able to reflect differences in structural configuration. Furthermore, when parameters such as skin thickness are varied, $D^{*}$ could also prove to be effective in distinguishing structural responses.

Although the current experimental campaign is still at an early stage, future work will quantify response heterogeneity, also by incorporating a damage model. For better understanding the potentiality of the $D^{*}$ index, a possible approach involves conducting both numerical and experimental campaigns where the impactor hits a single node or between two nodes, also varying the skin thickness. This would enable a deeper understanding of the local non-uniform behavior of the specimen, while the variation in skin thickness would further highlight the sensitivity of the index to stiffness. Furthermore, given that the heterogeneity also depends on the relationship between the impactor and the cell dimensions, future investigations will serve to emphasize these relationships. In this regard, when the cell size approaches zero, the specimen behaves as a homogeneous material. On the contrary, when the cell size is much larger than the strut diameter, pronounced heterogeneities emerge. In this context, the index appears to be the most sensitive indicator of structural heterogeneity.

## 6. Conclusions

This study aimed to investigate the low-velocity impact behavior of PLA-based BCC lattice structures, focusing on the influence of strut diameter and the role of local deformation mechanisms. Two configurations, differing solely in strut diameter (1.5 mm and 2 mm), were examined through a combined experimental and numerical approach. Drop-weight impact tests were carried out alongside finite element simulations using Ansys LS-DYNA (version 2023 R1).

To overcome the limitations of traditional global metrics (EA, SEA, MCF) in capturing localized deformation, a novel dimensionless index, $D^{*}$, was proposed. This index quantifies the ratio between local plastic indentation and global displacement, providing a more nuanced understanding of structural response. Moreover, by definition, the $D^{*}$ index proved to be an effective metric to capture the heterogeneity of the lattice structures. The results showed that the BCC structure with thinner struts, although characterized by lower stiffness, absorbed more energy, making it more suitable for non-structural applications—e.g., protective frames, battery housings, or sensor mounts in Unmanned Aerial Vehicles—where its reduced initial stiffness is offset by a more progressive deformation and a more stable EA, thus minimizing the risk of sudden failure.

Although the index shows limited generalizability in two separate scenarios—specifically, when the impactor strikes between four nodes, and when the configurations being compared share the same skin thickness—it still proves to be a promising tool under alternative impact conditions.

In conclusion, these results highlight the importance of integrating both global and local metrics when evaluating the impact performance of lattice structures, and demonstrate the potential of $D^{*}$ as a complementary tool for structural engineering assessment.

## Figures and Tables

**Table 1:** PLA characterization. Tensile test, mechanical properties.

Mechanical Property	Average Value	Standard Deviation
*E* [MPa]	3232.0	145.05
$\sigma_{yield}$ [MPa]	33.90	2.03
$\sigma_{max}$ [MPa]	42.39	1.77
$\varepsilon_{break}$	0.0528	0.0110

**Table 2:** Dimensional statistics (mean ± SD from raw measurements), and deviation from nominal values (Δ%) of the BCC1.5 and BCC2 samples.

Sample Category	$W_{M}\pm SD$ [mm]	$\Delta W_{nom}$ [%]	$L_{M}\pm SD$ [mm]	${\Delta L}_{nom}$ [%]	$t_{M}\pm SD$ [mm]	${\Delta t}_{nom}$ [%]
BCC1.5	100.05 ± 0.08	+0.05	149.82 ± 0.14	−0.12	12.15 ±0.03	+1.25
BCC2	100.13 ± 0.11	+0.13	150.30 ±0.08	+0.20	12.23 ±0.04	+1.92

**Table 3:** Coefficient of variation (CV%) of the BCC1.5 and BCC2 samples.

Sample Category	${CV}_{W}$ [%]	$CV_{L}$ [%]	$CV_{t}$ [%]
BCC1.5	0.08	0.09	0.29
BCC2	0.12	0.05	0.31

**Table 4:** Experimental values of $F_{max},$ $E_{max}$, $\delta_{max},$ $d_{1}$, $d_{2},EA$, SEA, $MCF\;and\;D^{*}$ after impact on BCC1.5 samples.

Sample ID	$F_{max}$ [N]	$E_{max}$ [J]	$\delta_{max}$ [mm]	$d_{1}$ [mm]	$d_{2}$ [mm]	EA [J]	SEA [J/g]	MCF [J/mm]	$D^{*}$
4BCCD1	673.60	3.06	7.27	−3.17	−3.30	2.72	0.04	0.37	0.44
4BCCD2	697.40	3.06	7.18	−2.16	−2.83	2.71	0.05	0.38	0.35
4BCCD3	734.90	3.06	7.05	−2.05	−2.28	2.56	0.04	0.36	0.31
4BCCD4	710.20	3.06	6.94	−3.01	−3.16	2.72	0.05	0.40	0.44
4BCCD5	683.00	3.06	7.11	−6.05	−5.95	2.69	0.04	0.38	0.84
4BCCD049	772.80	3.06	7.16	−2.82	−3.08	2.50	N/A	0.34	0.41

**Table 5:** Experimental values of $F_{max},$ $E_{max}$, $\delta_{max},$ $d_{1}$, $d_{2}$, EA, SEA, $MCF\;and\;D^{*}$ after impact on BCC2 samples.

Sample ID	$F_{max}$ [N]	$E_{max}$ [J]	$\delta_{max}$ [mm]	$d_{1}$ [mm]	$d_{2}$ [mm]	EA [J]	SEA [J/g]	MCF [J/mm]	$D^{*}$
4BCCD6	1040.60	2.98	4.80	−1.71	−1.57	2.30	0.03	0.48	0.34
4BCCD7	1096.00	2.98	4.90	−1.65	−1.62	2.51	0.03	0.51	0.33
4BCCD8	1000.60	3.01	4.86	−1.65	−1.56	2.38	0.03	0.49	0.33
4BCCD9	1029.60	3.01	4.79	−1.76	−1.78	2.41	0.03	0.50	0.37
4BCCD10	972.50	2.99	5.16	−1.99	−2.07	2.53	0.03	0.49	0.40
4BCCD051	937.20	2.99	5.41	−2.82	−2.93	2.52	N/A	0.46	0.53

**Table 6:** Experimental values mean EA, $SD\;EA,\;mean\;D^{*}\;and\;SD\;D^{*}$ for BCC1.5 and BCC2 samples.

Structure Category	Mean EA [J]	SD EA [J]	$Mean\;D^{*}$	$SD\;D^{*}$
BCC1.5	2.65	0.09	0.46	0.19
BCC2	2.44	0.09	0.38	0.07

**Table 7:** Mean experimental and numerical values of $F_{max},$ $E_{max}$, $\delta_{max},$ $\bar{d}$, EA, SEA, $MCF\;and\;D^{*}$ after impact on BCC1.5 and BCC2 samples.

Sample ID		$F_{max}$ [N]	$E_{max}$ [J]	$\delta_{max}$ [mm]	$\bar{d}$ [mm]	EA [J]	SEA [J/g]	MCF [J/mm]	$D^{*}$
**BCC1.5**	*FEM*	852.18	2.86	6.47	4.79	2.30	0.04	0.35	0.74
	*Mean EXP*	711.98	3.06	7.12	−3.32	2.65	0.04	0.37	0.46
**BCC2**	*FEM*	1030.90	2.81	5.29	3.67	2.25	0.03	0.42	0.70
	*Mean EXP*	1012.75	2.99	4.98	−1.92	2.44	0.03	0.49	0.38

## Data Availability

The original contributions presented in this study are included in the article. Further inquiries can be directed to the corresponding author.

## Appendix A

**Table A1:** Widths of the BCC1.5 and BCC2 samples.

Sample ID	$W_{1}$ [mm]	$W_{2}$ [mm]	$W_{3}$ [mm]	$W_{mean}$ [mm]
4BCCD1	100.07	100.07	100.15	100.09
4BCCD2	99.91	100.20	99.93	100.01
4BCCD3	100.04	99.96	100.24	100.08
4BCCD4	99.97	100.00	99.89	99.95
4BCCD5	100.00	100.00	99.99	99.99
4BCCD049	100.12	100.20	100.23	100.18
4BCCD6	99.99	100.00	100.02	100.00
4BCCD7	100.25	100.43	100.27	100.31
4BCCD8	100.40	99.94	100.33	100.22
4BCCD9	100.39	99.89	99.82	100.03
4BCCD10	100.37	100.09	99.84	100.10
4BCCD051	100.01	100.12	100.12	100.08

**Table A2:** Lengths of the BCC1.5 and BCC2 samples.

Sample ID	$L_{1}$ [mm]	$L_{2}$ [mm]	$L_{3}$ [mm]	$L_{mean}$ [mm]
4BCCD1	149.81	149.79	149.66	149.75
4BCCD2	149.89	149.65	149.72	149.75
4BCCD3	149.67	149.66	149.74	149.69
4BCCD4	149.87	149.83	149.79	149.83
4BCCD5	149.77	149.88	149.87	149.84
4BCCD049	150.06	150.11	150.06	150.07
4BCCD6	150.32	150.38	150.50	150.40
4BCCD7	150.21	150.38	150.45	150.35
4BCCD8	150.26	150.46	150.36	150.36
4BCCD9	150.16	150.32	150.38	150.29
4BCCD10	150.23	150.30	150.29	150.27
4BCCD051	150.10	150.19	150.20	150.16

**Table A3:** Thicknesses of the BCC1.5 and BCC2 samples.

Sample ID	$t_{1}$ [mm]	$t_{2}$ [mm]	$t_{3}$ [mm]	$t_{4}$ [mm]	$t_{5}$ [mm]	$t_{6}$ [mm]	$t_{mean}$
4BCCD1	12.14	12.13	12.07	12.14	12.02	12.20	12.12
4BCCD2	12.21	12.20	12.09	12.18	12.03	12.06	12.12
4BCCD3	12.22	12.20	12.00	12.20	12.04	12.00	12.11
4BCCD4	12.05	12.06	12.23	12.17	12.26	12.20	12.16
4BCCD5	12.09	12.07	12.21	12.20	12.26	12.25	12.18
4BCCD049	12.23	12.12	12.27	12.12	12.27	12.10	12.18
4BCCD6	12.36	12.12	12.30	12.23	12.33	12.36	12.29
4BCCD7	12.08	12.25	12.22	12.29	12.32	12.27	12.24
4BCCD8	12.07	12.20	12.22	12.18	12.33	12.38	12.23
4BCCD9	12.30	12.28	12.20	12.29	12.19	12.27	12.25
4BCCD10	12.09	12.12	12.13	12.18	12.24	12.28	12.17
4BCCD051	12.14	12.27	12.14	12.21	12.19	12.29	12.20

**Table A4:** Masses of the BCC1.5 and BCC2 samples.

Sample ID	Average Mass [g]
4BCCD1	58.69
4BCCD2	58.29
4BCCD3	58.10
4BCCD4	58.46
4BCCD5	58.15
4BCCD049	N/A *
4BCCD6	70.37
4BCCD7	70.67
4BCCD8	70.70
4BCCD9	71.41
4BCCD10	71.66
4BCCD051	N/A *

* The average mass values for 4BCCD049 and 4BCCD051 are not reported because these specific measurements were not taken during the first experimental campaign.
